# Characterization of the *In Situ* Ecophysiology of Novel Phylotypes in Nutrient Removal Activated Sludge Treatment Plants

**DOI:** 10.1371/journal.pone.0136424

**Published:** 2015-09-04

**Authors:** Simon Jon McIlroy, Takanori Awata, Marta Nierychlo, Mads Albertsen, Tomonori Kindaichi, Per Halkjær Nielsen

**Affiliations:** 1 Center for Microbial Communities, Department of Chemistry and Bioscience, Aalborg University, Aalborg, Denmark; 2 EcoTopia Science Institute, Nagoya University, Furo-cho, Chikusa-ku, Nagoya, Aichi, 464–8603, Japan; 3 Department of Civil and Environmental Engineering, Graduate School of Engineering, Hiroshima University, 1–4–1 Kagamiyama, Higashihiroshima, 739–8527, Japan; Australian Institute of Marine Science, AUSTRALIA

## Abstract

An in depth understanding of the ecology of activated sludge nutrient removal wastewater treatment systems requires detailed knowledge of the community composition and metabolic activities of individual members. Recent 16S rRNA gene amplicon surveys of activated sludge wastewater treatment plants with nutrient removal indicate the presence of a core set of bacterial genera. These organisms are likely responsible for the bulk of nutrient transformations underpinning the functions of these plants. While the basic activities of some of these genera *in situ* are known, there is little to no information for the majority. This study applied microautoradiography coupled with fluorescence *in situ* hybridization (MAR-FISH) for the *in situ* characterization of selected genus-level-phylotypes for which limited physiological information is available. These included *Sulfuritalea* and A21b, both within the class *Betaproteobacteria*, as well as Kaga01, within sub-group 10 of the phylum Acidobacteria. While the *Sulfuritalea* spp. were observed to be metabolically versatile, the A21b and Kaga01 phylotypes appeared to be highly specialized.

## Introduction

Wastewater treatment using activated sludge represents one of the largest biotechnology industries in the world. These systems employ a diverse consortium of microbes primarily for the removal of carbon, nitrogen and phosphorus from wastewater streams. Removal of such nutrients is important for the prevention of the eutrophication of aquatic ecosystems receiving the treated water [1]. An in depth understanding of the ecology of these biotechnological systems is key for their optimal design and efficient operation [2]. Important to this goal will be the characterization of the individual contribution of each bacterial genus-level-taxon to system function [3].

Several long-term surveys of full-scale treatment plants, with both quantitative fluorescence *in situ* hybridization (qFISH) [2,4] and 16S rRNA gene sequencing [5,6], have aimed to elucidate the microbial community composition of these systems. An amplicon-sequencing based survey of 13 Danish full-scale plants with nutrient removal observed that a relatively small number of genus-level-taxa (64) constitute a large percentage (approx. 70% read abundance on average) of the community. These abundant core genera are therefore likely collectively responsible for the bulk of the nutrient transformations that underpin system function [6]. The subsequent goal will be to systematically characterize each of the abundant organisms and add this information to public resources such as the MiDAS database for wastewater treatment systems [3]. Most of these groups have no cultured representatives and for those that do the isolates do not always represent the abundant environmental strains [7]. Moreover, microorganisms appear to be much more specialized *in situ* than when grown in pure culture [8]. *In situ* methods, such as FISH in combination with microautoradiography (MAR) [9], are therefore essential in determining the ecology of these organisms. While *in situ* information is available for some there is little to no information for the majority, and thus no indication as to their relevance to the ecology of these systems.

This study applied MAR-FISH based methods to characterize a selection of the abundant core phylotypes identified by Saunders et al. [6] for which limited information is available about their ecophysiology in activated sludge. These included the genus *Sulfuritalea* and the novel genus-level-taxon A21b, both within the class Betaproteobacteria, as well as a genus-level-taxon, within sub-group 10 of the phylum Acidobacteria, known as Kaga01. All were reported to be among the top 10 genus-level-taxa by median read abundance [6].

## Materials and Methods

### Phylogenetic analysis and probe design

Phylogenetic placement of representative 16S rRNA gene sequences (generated by Saunders et al. [6]) and the design of FISH probes for selected groups were performed with the ARB software package [10]. Potential probes were assessed *in silico* with the mathFISH software for hybridization efficiencies of target, and potentially weak non-target, matches [11]. Unlabeled helper probes [12] were designed for calculated inaccessible regions. Unlabeled competitor probes were also designed for single base mismatched non-target sequences [13]. The existence of non-target indel sequences were identified with the Ribosomal Database Project (RDP) PROBE MATCH function [14] and assessed with the LOOPOUT program [15]. Probe validation and optimization was based on generated formamide dissociation curves [16], where fluorescent intensities of at least 50 cells were measured with ImageJ software (National Institutes of Health, Maryland, USA). Calculated average values were compared for hybridization buffer formamide concentration over a range of 5–50% (v/v) with 5% increments (data not shown). As no isolates were available for probe target groups, activated sludge from four separate full-scale WWTPs was used to optimize the probes. Where available, weak base-mismatch non-target axenic cultures were also used for probe assessment (see **Table 1**).

**Table 1:** Probes designed and optimized in this study.

Probe	*E*. *coli* pos.	Target group	*Coverage* ^§^	Non-target hits	Sequence (5’-3’)	Validated against^⌘^	Required^#^	[FA]%^Φ^
**Acido-1162**	**1162–1179**	***Acidobacteria* sub-group 10**	**283/370 (76%)**	**6**	**TCC TCC CCG ATT TCC GGG**	**Full-scale sludge**	**NA**	**25**
Acido-1162c1	1162–1179	Competitor for Acido_1162	NA	NA	TCC TCC CCG TTT TCC GGG	-	ND	NA
Acido-1162h1	1180–1201	Helper for Acido_1162	NA	NA	GGA CTT GAC GTC ATC CCC RCC Y	Full-scale sludge	No	NA
**Acido-819**	**819–836**	**ABS-19**	**67/120 (56%)**	**0**	**GCG ACA CCG AGC ACC CAT**	**Full-scale sludge**	**NA**	**25**
		*p7o14 (ABS-19 sub-group)*	*58/88 (66%)*	*NA*				
		*Kaga01 (ABS-19 sub-group)*	*9/9 (100%)*	*NA*				
Acido-819c1	819–836	Competitor for Acido_819	NA	NA	GCG ACA CCT AGC ACC CAT	*Phaselicystis flava* DSM21295^T^	Yes	NA
Acido-819c2	819–836	Competitor for Acido_819	NA	NA	GCG ACA CCG AGT ACC CAT	-	ND	NA
Acido-819c3	819–836	Competitor for Acido_819	NA	NA	GCG ACA CCG AGC ATC CAT	-	ND	NA
Acido-819h1	837–854	Helper for Acido_819	NA	NA	ACC GCA GGG GTC GAT ACC C	Full-scale sludge	No	NA
Acido-819h2	796–818	Helper for Acido_819	NA	NA	CGT TTA CAG CGT GGA CYA CCA GG	Full-scale sludge	No	NA
**SCI84–971**	**971–989**	**SC-I-84**	**314/377 (83%)**	**18**	**GGT AGG TAA GGT TGT TCG C**	**Full-scale sludge**	**NA**	**25**
SCI84–971c1	971–989	Competitor for SCI84_971	NA	NA	GGT AGG TAA GGT TYT TCG C	*Achromobacter ruhlandii* DSM653^T^	No	NA
SCI84–971h1	946–970	Helper for SCI84_971	NA	NA	GTT GCA TCG AAT TAA TCC ACA TCA T	Full-scale sludge	Yes	NA
**SCI84–829**	**829–851**	**A21b**	**23/25 (92%)**	**2**	**CAG AGA TCG CTC CCC GAA CAC C**	**Full-scale sludge**	**NA**	**30**
SCI84–829h1	802–827	Helper for SCI84_829	NA	NA	AGT GAT CAT CGT TTA GGG CGT GGA CT	Full-scale sludge	No	NA
SCI84–829h2	853–878	Helper for SCI84_829	NA	NA	ATC ACT TCA CGC GTT AGC TWC GGT AC	Full-scale sludge	No	NA

**§** Coverage of groups as defined in MiDAS database (Release 1.20)[3]. Values given as group hits/ group totals. Percentage values are given in parenthesis;

⌘ For optimization with ‘Full-scale sludge’ samples taken from the Odense North East, Odense North West, Ejby Mølle and Randers BNR WWTPs were used. In each case optimization with the different plants gave similar dissociation profiles (data not shown);

# Requirement of accessory probes: If available, isolates with single-base mismatches to the probe were used to assess the requirement for a competitor probe. Competitor probes perfectly match the target site in the listed isolate. If the mismatch alone is enough to prevent non-target binding of the labeled probe, at the recommended formamide concentration, then the competitor probe is assessed as not being required (note: it is recommended that competitor probes are included for un-validated mismatches); helper probes assessed on ability to improve fluorescence signal of the associated labeled probe. NA = Not applicable; ND = Not determined;

**Φ** Determined optimal formamide concentration for use in FISH hybridizations.

### Fluorescence *in situ* hybridization (FISH)

Selected activated sludge samples were obtained from the aerobic tanks of full-scale WWTPs as part of the MiDAS FISH survey [4]. Sample fixation and FISH were performed essentially as described by Daims et al. [16]. Probes were applied, with recommended competitors and helpers, at the stringency conditions given in **Table 1**or their original publications. The NON-EUB nonsense probe was used as a negative hybridization control [17]. Both the 3’ and 5’ ends of oligonucleotide probes were labeled, with 5(6)-carboxyfluorescein-*N*-hydroxysuccinimide ester (FLUOS) or with the sulfoindocyanine dyes (Cy3 and Cy5) (DOPE-FISH [18]), in order to improve the *in situ* fluorescent signal to background ratio. Microscopic analysis was performed with either an Axioskop epifluorescence microscope (Carl Zeiss, Oberkochen, Germany) or a LSM510 Meta laser scanning confocal microscope (Carl Zeiss).

### Microautoradiography (MAR)-FISH

Activated sludge was sampled from aeration tanks at Randers WWTP (56.453767° N, 10.070249° E) (sampled January 7^th^, 2014) and Ejby Mølle WWTP (55.399534° N, 10.414787° E) (sampled January 22^nd^, 2014) and transported overnight. Both plants are configured for biological phosphorus removal and have stable performance. The Randers WWTP is a recirculation plant with side stream hydrolysis and the Ejby Mølle WWTP has an alternating configuration (Biodenipho). For further details on the plants assessed in this study see Mielczarek et al. [19]. MAR incubations were performed immediately upon arrival in the laboratory. Sampling was carried out with the permission of Randers Spildevand A/S and VandCenter Syd A/S.

The MAR-FISH procedures were carried out essentially as described in Nielsen and Nielsen [20]. Radiolabeled compounds used were: [^14^C]-propionate, [^14^C]-butyrate, [^3^H]-amino acid mix, [^3^H]-ethanol, [^3^H]-glycerol, [^3^H]-N-acetyl glucosamine, (American Radiolabeled Chemicals Inc., Saint Louis MO, USA); [^3^H]-acetate, [^14^C]-pyruvate, [^3^H]-glucose (PerkinElmer Inc., Waltham MA, USA); [^3^H]-oleic acid (Amersham Bioscience, Denmark).

Prior to MAR incubations, the activated sludge was aerated for 1 h to remove traces of organics present. For all anaerobic incubations an additional 1 h anaerobic pre-incubation was performed to remove traces of oxygen and NOx present. For MAR incubations, pre-incubated mixed liquor was diluted to 1 gSS/L with sludge supernatant and transferred to 11 ml serum bottles. Unlabeled and labeled substrates were added to give final concentrations of 2 mM and 10 μCi/ml, respectively. For anaerobic incubations oxygen was removed by repeated evacuation of the headspace and subsequent injection of O_2_-free N_2_ prior to substrate addition. Bottles were sealed with thick rubber stoppers and incubated for 3 h. In order to better assess true denitrification, anaerobic incubations with selected substrates were supplemented with 0.5 mM nitrite, as many non-denitrifiers are able to reduce nitrate to nitrite [21]; acknowledging that this will not cover denitrifiers unable to directly utilize nitrite. For incubations assessing the use of nitrite as an electron acceptor, the supernatant nitrite concentration was monitored during the incubation period using Quantofix Nitrate Nitrite strips (Macherey-Nagel, Düren, Germany) and readjusted to approx. 0.5 mM anaerobically to prevent exhaustion. Incubations using pasteurized mixed liquor (70°C, 10 min) were performed as negative controls for all experiments.

Following MAR incubations, the cells were fixed for 3h with cold 4% [w/v] paraformaldehyde (final concentration) and the fixed samples were washed 3 times with sterile filtered tap water. Aliquots of 20 μl of the biomass were gently homogenized between glass coverslips. Following FISH, slides were coated with Ilford K5D emulsion (Polysciences, Inc., Warrington, PA, USA), exposed in the dark for 10 days and developed with Kodak D-19 developer.

## Results

### FISH probe design and optimization

**Fig 1:**
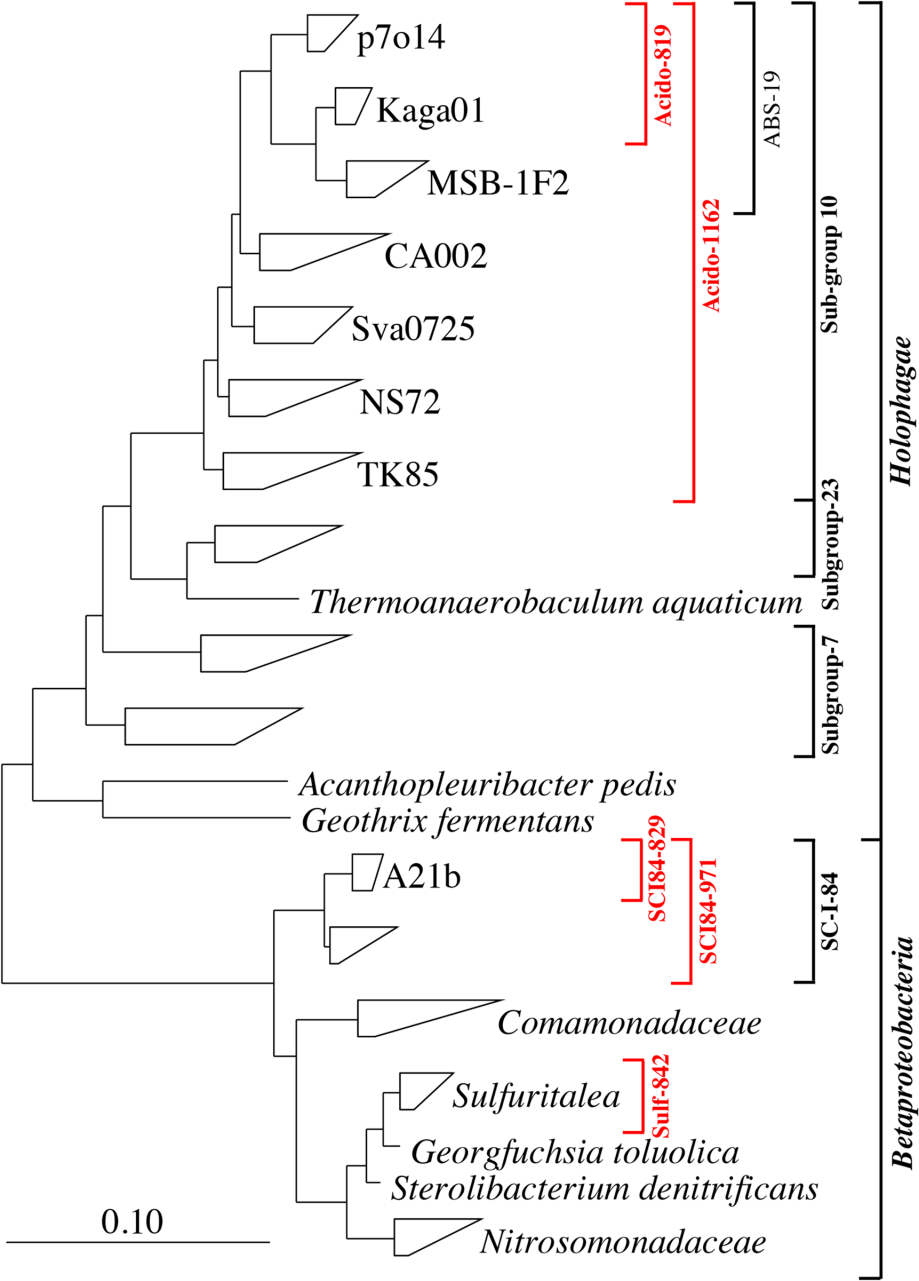
Maximum-likelihood (PhyML) 16S rRNA gene phylogenetic tree of target groups and selected related sequences. All sequences are >1200 bp in length. Phylogenetic classification is taken from the MiDAS database (Release 1.20), which is a version of the SILVA database (Release 119; [23]) curated for activated sludge sequences [3]. Clades represent 5–15 selected sequences. Probe coverage for probes applied in this study is shown in red. For the Sulf842 this represents the overlap in coverage for the Sulf842 and Sulf432 probes. The scale bar represents substitutions per nucleotide base.

**Fig 2:**
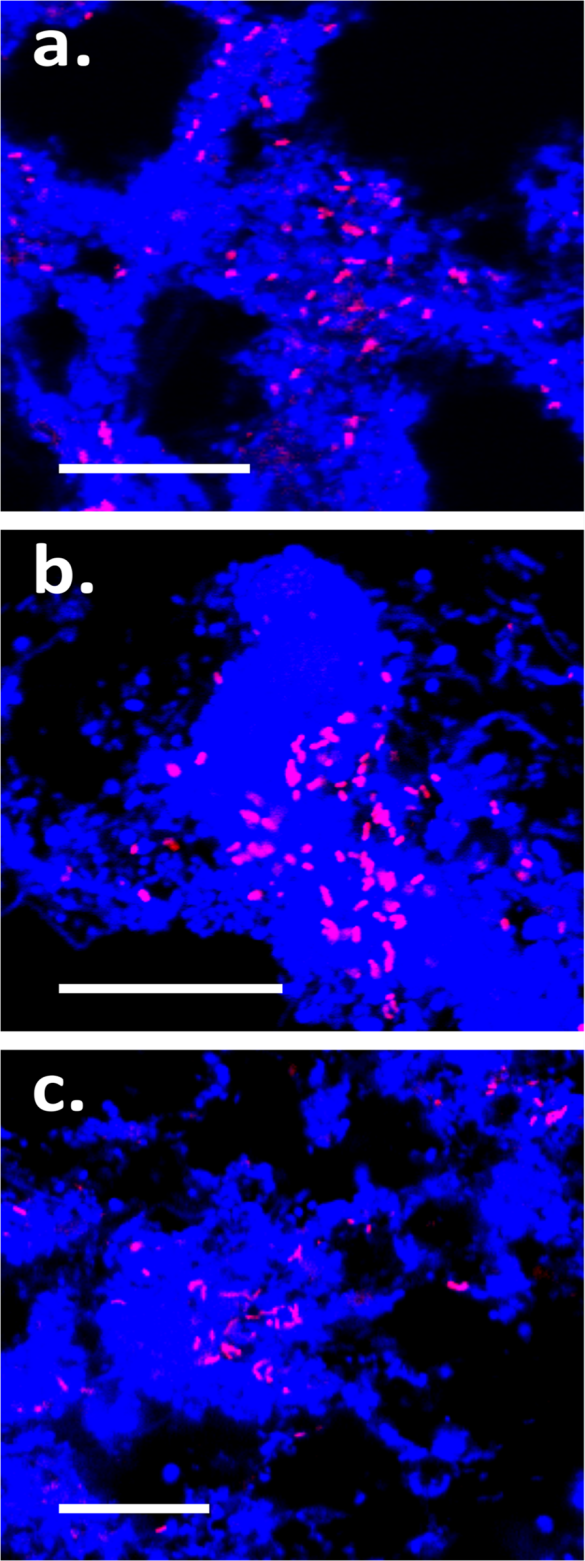
Composite FISH micrographs of target phylotypes in full-scale sludge. (**a**) *Sulfuritalea*-related (Sulf-842); (**b**) A21b (SCI84–829); (**c**) ABS-19 (Acido-819). Target cells in FISH micrograph overlays appear magenta [target probe (Cy3 = red) + EUBmix (Cy5 = blue)] and non-target blue (EUBmix only). Scale bars represent 20 μm.

Three of the top ten most abundant genus-level-taxa, from a recent survey of 13 WWTPs in Denmark [6], were selected for FISH-based characterization. These include the betaproteobacterial genera Ellin_Unk01 and *Sulfuritalea*, and the genus Sva0725 of the phylum Acidobacteria. These were originally classified with the Greengenes taxonomy (version October 2012) [22], but in the updated MiDAS taxonomy (version 1.20) they classify as the genera A21b, *Sulfuritalea* and Kaga01, respectively [3]. Details of FISH probe optimization and recommendations for their application are given in **Table 1.** Probe coverage and phylogeny are shown in **Fig 1**. FISH images of the selected phylotypes are shown in **Fig 2**.

The previously designed Sulf842 probe was applied to cover the *Sulfuritalea* spp. [24]. The specificity of the Sulf842 probe was confirmed for the Ejby Mølle and Randers WWTP communities by the recommended application of the Sulf431 probe labeled with a different fluorochrome (see McIlroy et al. [24]).

In this study the SCI84–829 was designed and optimized for coverage of the MiDAS defined genus-level-taxon A21b. The probe hybridized with small rod shaped cells (typically approx. 0.6 × 1.5 μm) that were mostly present as single cells, but were also observed as loosely associated clusters (see **Fig 2B**). Quantitative FISH analysis suggests that members of the genus are not abundant in full-scale communities (< 1% of the biovolume (**S1 Table**)). The SCI84–971 was designed to cover the candidate order SC-I-84, containing the A21b phylotype, to allow higher confidence in the specificity of the SCI84–829 probe. Cells hybridising with the SCI84–829 probe showed good overlap with the SCI84–971 probe, which supports a high specificity of the former probe.

Probes specific for the Kaga01 were not found. However, the Acido-819 probe was designed to cover both Kaga01 and p7o14, which are both genus-level taxa within the family-level-taxon ABS-19. Sequences classifying to the genus p7o14 are rarely detected in full-scale sludges (see **Table 2**), thus Acido-819 probe positive cells in activated sludge likely belong to the Kaga01 genus. The probe hybridized with small rods (typically approx. 0.5 × 1.4 μm), present as dispersed single cells in full-scale sludges (see **Fig 2C**). Positive cells constituted up to 2% of the community biovolume in full-scale systems (see **S1 Table**). A hierarchical probe set was also applied to better assess specificity. Good overlap was observed for the Acido-819 probe and the broader newly designed Acido-1162, targeting the order sub-group 10 of the Acidobacteria, and the SS_HOL1400 probe [25], targeting the phylum Acidobacteria.

**Table 2:** Distribution of selected phylotypes in full-scale WWTPs.

Phylotype	Abundance (%)				
	Median	Q25	Q75	Min	Max
*Sulfuritalea*	0.4	0.2	0.7	<0.1	2.4
A21b	0.1	<0.1	0.1	-	0.4
Kaga01 (ABS-19)	0.1	0.1	0.2	<0.1	1.0
p7o14 (ABS-19)	-	-	-	-	<0.1

Data is sourced from the MiDAS 16S amplicon survey of 20 full-scale WWTPs in Denmark over 8 years (n = 396)(Albertsen, M., Nielsen, P.H. et al., unpublished—for details see McIlroy et al. [3]).

### Distribution of the phylotypes

The genera characterized represent three of the top ten genera, by median abundance (all > 1%), of a previous 16S rRNA gene based survey of 13 full-scale nutrient removal WWTPs [6]. However, the recent 8-year MiDAS survey of 20 Danish full-scale treatment plants suggested lower abundances (see **Table 2**). Quantitative FISH analyses performed with the probes designed in this study better supported the values of the MiDAS survey (see **S1 Table**), suggesting they were overestimated in the previous study. One key difference between these studies was the use of primers targeting different regions of the 16S rRNA gene. The v4 and v1–3 regions were used for the surveys of Saunders et al. [6], and the MiDAS, respectively. However, recent analyses comparing the application of these primer sets to activated sludge found no substantial difference in the calculated abundances of the three phylotypes investigated in this study [26]. The only other major difference between the MiDAS survey and study of Saunders et al. [6] is the DNA extraction method applied. Both studies applied the Fast DNA Soil kit (MP Biomedicals, USA), but the former used an extended bead beating time for cell lysis while the latter used hot phenol (at 80°C). The influence of DNA extraction on the resulting community composition is well studied for activated sludge samples (see Albertsen et al. [26]) and is likely largely responsible for the previous overestimation of the phylotypes analysed here.

### *In situ* physiology

**Fig 3:**
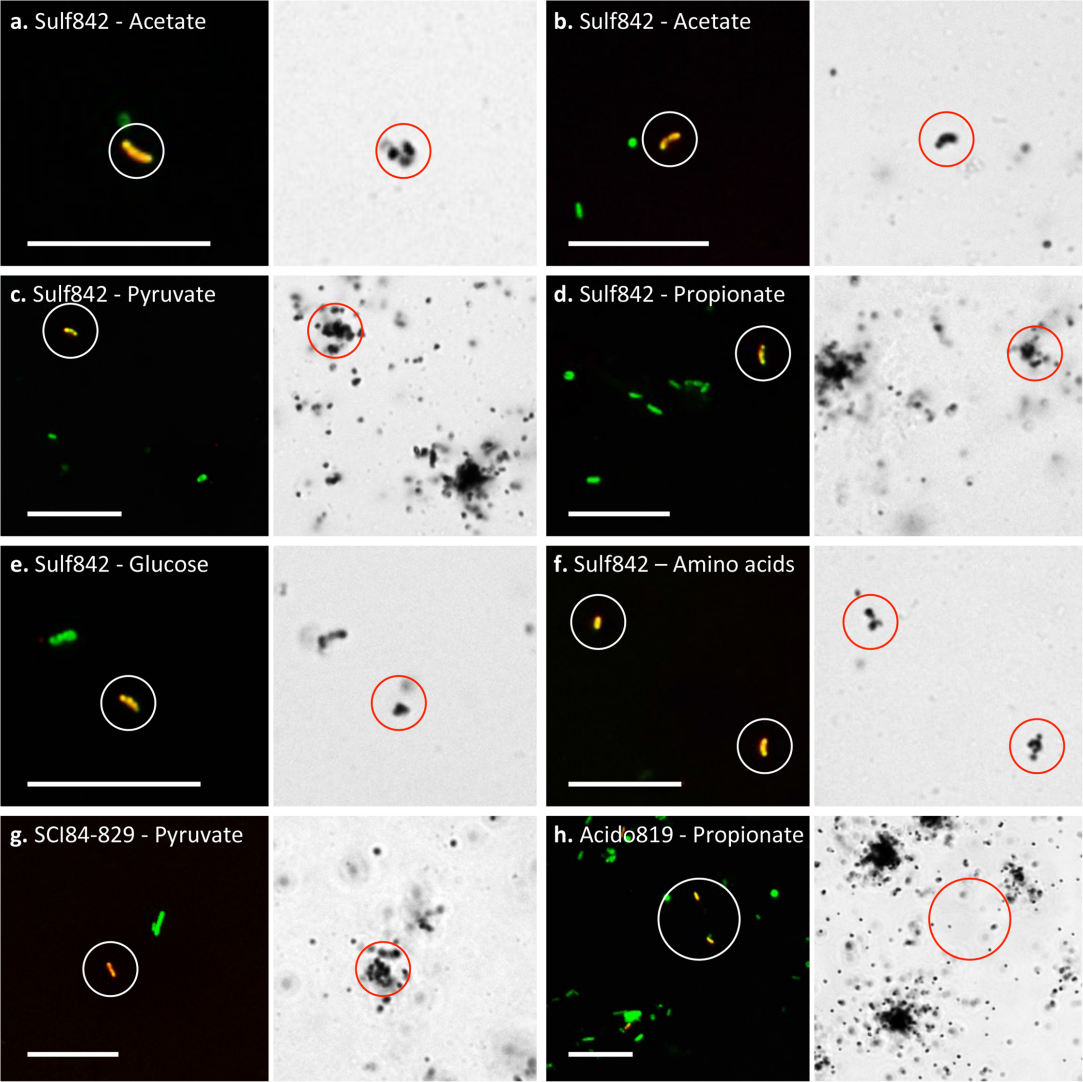
Selected FISH and corresponding bright-field MAR micrographs. FISH probes and MAR substrate are indicated for each image set. Target cells in FISH micrograph overlays appear yellow [target probe (Cy3 = red) + EUBmix (FLUOS) = green] and non-target green (EUBmix only). Circles indicate location of FISH-positive cells. Black silver granules indicate positive MAR signal: **a.-g.** show positive MAR signal for cells hybridizing with the specific FISH probe; **h.** shows a negative MAR signal. Images represent incubations performed under either aerobic (**a. and c.-h.**) or anaeorbic incubation conditions (**b.**) with biomass from the Randers (**a., b., d. and f.-h.**) or the Ejby Mølle (**c. and e.**) WWTPs. Scale bars represent 10 μm.

The *Sulfuritalea* spp. were able to assimilate pyruvate, acetate and amino acids, while the uptake of propionate and glucose varied between plants (**Fig 3A–3F; Table 3**). With the exception of propionate, all substrates could be assimilated under both aerobic and anaerobic conditions. Butyrate, oleate, glycerol, ethanol and *N*-acetylglucosamine were not assimilated by the genus in either WWTP (**Table 3**).

**Table 3:** Summary of MAR-FISH results for carbon uptake profiles.

Substrate	*e-* acceptor*	Phylotype (FISH probe)					
		*Sulfuritalea* (Sulf-842)		A21b (SCI84–829)		ABS-19 (Acido-819)	
		EM	RA	EM	RA	EM	RA
^14^C-Pyruvate	O_2_	++	++	++	++	−	−
^14^C-Pyruvate	NO_2_^-^	++	++	+	±	−	−
^14^C-Pyruvate	−	++	++	+	±	−	−
^3^H-Acetate	O_2_	±	+	−	−	−	−
^3^H-Acetate	NO_2_^-^	±	+	−	−	−	−
^3^H-Acetate	−	±	+	−	−	−	−
^14^C-Propionate	O_2_	−	++	−	−	−	−
^14^C-Propionate	−	ND	−	−	−	−	−
^14^C-Burtyrate	O_2_	−	−	−	−	−	−
^14^C-Burtyrate	−	ND	ND	−	−	−	−
^3^H-Oleate	O_2_	−	−	−	−	−	−
^3^H-Oleate	−	ND	ND	−	−	−	−
^3^H-Amino acids	O_2_	++	++	−	−	−	−
^3^H-Amino acids	NO_2_^-^	++	++	−	−	−	−
^3^H-Amino acids	−	++	++	−	−	−	−
^3^H-Glucose	O_2_	±	−	−	−	−	−
^3^H-Glucose	−	+	−	−	−	−	−
^3^H-NAG	O_2_	−	−	−	−	−	−
^3^H-NAG	−	ND	ND	−	−	−	−
^3^H-Glycerol	O_2_	−	−	−	−	−	−
^3^H-Glycerol	−	ND	ND	−	−	−	−
^3^H-Ethanol	O_2_	−	−	−	−	−	−
^3^H-Ethanol	−	ND	ND	−	−	−	−

Data represent the proportion of 50 FISH-positive cells that are also MAR positive: −, ≤ 10%; ±, 11–30%; +, 30–60%; ++, > 60%. ND = Not determined. Results are given for both WWTPs: **EM** = Ejby Mølle; **RA** = Randers.

*Electron acceptors: ‘**O2’** = aerobic conditions; ‘**NO**_**2**_^**-**^ **’** = anaerobic conditions with nitrite added; ‘**−**’ = None added, anaerobic conditions.

Of the substrates tested, members of the A21b phylotype were only able to assimilate pyruvate (**Table 3; Fig 3G**). Uptake of pyruvate under anaerobic conditions was detected, indicating that it is possibly stored and/or fermented or that an alternate unidentified residual electron acceptor is utilized. Addition of nitrite under anaerobic conditions did not appear to increase substrate uptake, thus suggesting an inability for denitrification.

ABS-19 species were unable to assimilate any of the substrates tested (**Table 3, Fig 3H**). Assessment of several autotrophic activities gave inconclusive results (see **S1 Text**).

## Discussion

This study applied MAR-FISH for the preliminary characterization of the *in situ* behaviour of three phylotypes suggested as abundant members of the activated sludge community. Quantitative FISH values, supported by more recent surveys with optimized protocols [3,26], suggest that *Sulfuritalea*, A21b and Kaga01 taxa were previously overestimated [6]. With widespread application of 16S rRNA gene amplicon sequencing to characterize bacterial communities it is important to acknowledge that the abundance values obtained are estimations at best. The observations of this study highlight the need for DNA extraction and amplification independent approaches, such as the FISH analyses conducted here, in the validation of profiling methods.

Despite being present at lower abundances than originally reported, the *Sulfuritalea* are still among the most 50 abundant genera in full-scale plants in Denmark [3]. Other than a reported ability for denitrification with amino acids as carbon source [24], little was known about the *in situ* physiology of the *Sulfuritalea* spp. in activated sludge. This study found these organisms to have broad substrate specificity, including short chain fatty acids, amino acids and perhaps sugars. Their ability to assimilate acetate under anaerobic conditions (**Fig 3B**) suggests that they are able to utilize an unidentified residual terminal electron acceptor. One possibility is Fe(III) [27], a hypothesis supported by its close relatedness of the genus to the iron reducing *Georgfuchsia toluolica* [28] (see **Fig 1**). However, such activities remain to be tested for members of the genus *in situ*, with the removal of residual ferric iron from the activated sludge being problematic. *Sulfuritalea hydrogenivorans* ^T^, the sole described species of the genus, is able to use molecular hydrogen and thiosulphate as sole electron donors with nitrate as the terminal electron acceptor under anaerobic conditions. Such ability for the species in activated sludge could not be confirmed in this study (see **S1 Text**) and the potential for mixotrophic growth remains to be demonstrated.

While the substrate uptake profiles were similar for the target phylotypes in both plants assessed in this study, there were differences in the uptake of glucose and propionate for the *Sulfuritalea* spp.. Such an observation is not unusual and, while the genus is considered a reasonably good phylogenetic unit for study [3], it is worth acknowledging that different members of the same genus may occupy slightly different niches.

Members of the genus-level-taxa A21b and Kaga01 appeared much more specialized than the *Sulfuritalea*. The A21b phylotype was only able to assimilate pyruvate, which it may ferment, while carbon uptake for the Kaga01 FISH-positive cells was not detected. However, it is unlikely that the species are not metabolically active *in situ* as they are reported to be growing in the system, and not simply washed in with the influent [6]. The observed lack of activity may be due to the failure of the applied experimental conditions to replicate the optimal conditions experienced by the species in the full-scale sample.

While members of the class Betaproteobacteria are well studied in activated sludge, little is known of the role of the phylum Acidobacteria. Members of the phylum were first detected in WWTPs with FISH by Ludwig et al. [29], but to our knowledge this is the first attempt to characterize their *in situ* behaviour in activated sludge. The phylum is phylogenetically diverse and relatively poorly studied, with few characterized isolates [30]. The Kaga01 phylotype belongs to the order ‘sub-group 10’ of the Acidobacteria (**Fig 1**), for which there are currently no cultured isolates.

This study has provided preliminary characterization of the potential activities of the genera *Sulfuritalea* and A21b. Further classification is required to elucidate the ecology of the phylotypes of this study; especially for the A21b and Kaga01, which appear to have relatively specialized metabolisms in sludge. Given the labor intensive nature of *in situ* methods, such as MAR-FISH, obtaining genomes from these uncultured organisms [31] will allow a more targeted approach to characterize their ecology in the future. For example, we recently applied MAR-FISH to assess the genome annotation-derived potential of the uncultured “*Ca*. Competibacter spp.” to ferment glucose [32]. The class and genus level FISH probes described in this study will be essential tools for future analyses of these organisms in both activated sludge and other environments.

## Supporting Information

S1 Table Abundance estimations for the selected phylotypes. § Egå, Viby, Aars, Randers and Hjørring have biological nitrogen and phosphorus removal; Esbjerg East nitrogen removal only. * Taken from the MiDAS amplicon survey. Plants and sample times selected for qFISH gave the highest abundances for the phylotypes in the extended MiDAS amplicon survey (53 plants). **Quantitative FISH (qFISH) values represent a percentage of the EUBmix positive cells that also hybridize with the specific probe (biovolume %) ± the standard deviation. Each value is determined from the analysis of 20 FISH micrographs (at 630x magnification) using the Daime image analysis software.
(DOCX)

S1 Text Labeled ^14^CO_2_-MAR for the detection of heterotrophic and autotrophic activities. Labeled ^14^CO_2_ uptake MAR electron acceptor/donor combinations tested (Table A). FISH and corresponding bright-field MAR micrographs for ^14^CO_2_ incubations (Figure A).
(PDF)
