# Supplementary material for: Characterization of the In Situ Ecophysiology of Novel Phylotypes in Nutrient Removal Activated Sludge Treatment Plants
Source: PLoS One. 2015 Sep 4;10(9):e0136424. doi: 10.1371/journal.pone.0136424 (PMC4560404; doi:10.1371/journal.pone.0136424)
Supplement: S1 Table — § Egå, Viby, Aars, Randers and Hjørring have biological nitrogen and phosphorus removal; Esbjerg East nitrogen removal only. * Taken from the MiDAS amplicon survey. Plants and sample times selected for qFISH gave the highest abundances for the phylotypes in the extended MiDAS amplicon survey (53 plants). **Quantitative FISH (qFISH) values represent a percentage of the EUBmix positive cells that also hybridize with the specific probe (biovolume %) ± the standard deviation. Each value is determined from the analysis of 20 FISH micrographs (at 630x magnification) using the Daime image analysis software. (DOCX) [file pone.0136424.s001.docx]

**Table S1:**

**Characterisation of the *In Situ* Ecophysiology of Novel Phylotypes in Nutrient Removal Activated Sludge Treatment Plants**

Simon Jon McIlroy, Takanori Awata, Marta Nierychlo, Mads Albertsen, Tomonori Kindaichi and Per Halkjær Nielsen

**Table S1.** Abundance estimations for the selected phylotypes

| **Phylotype (FISH probe)** | **Full-scale WWTP^§^** | **Sample date** | **Abundance %** | |
| --- | --- | --- | --- | --- |
|  |  |  | **Amplicon*** | **qFISH**** |
| A21b | Esbjerg East | 19-08-2006 | 0.5 | < 1 |
| (SCI84-829) | Egå | 28-10-2008 | 0.4 | < 1 |
|  | Viby | 19-08-2007 | 0.4 | < 1 |
| Kaga01 | Aars | 19-08-2008 | 1.4 | 2 ± 1 |
| (Acido-819) | Randers | 04-02-2006 | 0.9 | 2 ± 1 |
|  | Hjørring | 19-08-2009 | 1.0 | 2 ± 1 |

§ Egå, Viby, Aars, Randers and Hjørring have biological nitrogen and phosphorus removal; Esbjerg East nitrogen removal only. * Taken from the MiDAS amplicon survey (for details see [1]). Plants and sample times selected for qFISH gave the highest abundances for the phylotypes in the extended MiDAS amplicon survey (53 plants). **Quantitative FISH (qFISH) values represent a percentage of the EUBmix positive cells that also hybridize with the specific probe (biovolume %) ± the standard deviation. Each value is determined from the analysis of 20 FISH micrographs (at 630x magnification) using the Daime image analysis software [2].

**References**

1. McIlroy S, Saunders AM, Albertsen M, Nierychlo M, McIlroy B, Hansen AA, et al. MiDAS: the field guide to the microbes of activated sludge. Database. 2015; 2015: bav062.

2. Daims H, Lücker S, Wagner M. daime, a novel image analysis program for microbial ecology and biofilm research. Env. Microbiol. 2006; 8: 200–213.
