# Supplementary material for: Characterization of the In Situ Ecophysiology of Novel Phylotypes in Nutrient Removal Activated Sludge Treatment Plants
Source: PLoS One. 2015 Sep 4;10(9):e0136424. doi: 10.1371/journal.pone.0136424 (PMC4560404; doi:10.1371/journal.pone.0136424)
Supplement: S1 Text — Labeled 14CO2 uptake MAR electron acceptor/donor combinations tested (Table A). FISH and corresponding bright-field MAR micrographs for 14CO2 incubations (Figure A). (PDF) [file pone.0136424.s002.pdf]

S1 Text:

Characterization of the *In Situ* Ecophysiology of Novel Phylotypes in Nutrient Removal Activated Sludge Treatment Plants

Simon Jon McIlroy, Takanori Awata, Marta Nierychlo, Mads Albertsen, Tomonori Kindaichi and Per Halkjær Nielsen

Labeled $^{14}CO_2$-MAR for the detection of heterotrophic and autotrophic activities

MAR uptake profiles for the A21b and ABS-19 phylotypes revealed that, of the substrates assessed, the former was only able to assimilate pyruvate, and the latter was unable to assimilate detectable amounts of any. HetCO_2 MAR-FISH [1] was therefore applied with a complex carbon source to assess heterotrophic activity of these phylotypes. Labeled $^{14}CO_2$-MAR was also used to assess various autotrophic activities. Pure culture studies of the only isolate of the *Sulfuritalea*, *S. hydrogenivorans*^{T}, demonstrates an ability of the species to oxidize both molecular hydrogen and thiosulphate under anaerobic conditions with nitrate as electron acceptor. Additional MAR-FISH experiments were designed to assess if the related members of the genus present in full-scale sludge performed such activities.

Materials and Methods

Utilization of unlabeled electron acceptors/donors was assessed with the inclusion of NaH[$^{14}CO_2$] to detect CO_2 incorporation during heterotrophic or autotrophic growth [1,2]. In order to remove CO_2 species, mixed liquor was acidified to pH 5 using HCl, allowed to stand for 20 min, sparged with N_2 for 1 h and then adjusted to pH 7 with NaOH [2]. NaH[$^{14}CO_2$] (American Radiolabeled Chemicals Inc., Saint Louis MO, USA) was added to a final concentration of 20 µCi/ml. Unlabeled electron donors/acceptors utilized included: ammonia (1 mM), nitrite (0.5 mM), nitrate (2 mM), thiosulfate (2 mM), formate (2 mM), H_2 (1% [v/v]) and a complex carbon mix consisting of 0.16 g/L yeast extract, 0.16 g/L meat extract and 0.16 g/L casein hydrolysate. A summary of the electron acceptor and donor combinations is given in **Table A**. Incubation length was 5 h. The first washing step was performed with 0.1 M citrate buffer (pH 3). All other conditions and steps were the same as detailed in the main text.

Quantitative MAR was performed for selected conditions with a custom-made macro (MARQuant) for the Image J software [3]. The script calculated the average number of silver grains per cell, correcting for background MAR signal, for at least 50 cells. Statistical analysis was performed for the Sulf-842 probe-defined population to assess if selected electron donor and acceptor combinations gave a significantly higher level of activity than anaerobic incubation with $^{14}CO_2$ alone (two sample t-test, $P < 0.05$).

Table A. Labeled $^{14}CO_2$ uptake MAR electron acceptor/donor combinations tested

e- donor*	*e*- acceptors**
Complex carbon	O_2<br>NO_2^-<br>N.A.
HCO_2^-	O_2
NH_3	O_2
NO_2^-	O_2
H_2	O_2<br>NO_2^-<br>N.A.
$S_2O_3^{2-}$	O_2<br>NO_2^-<br>N.A.
N.A.	NO_3^-
N.A.	N.A.

***Electron donors: Complex carbon** = consisting of yeast extract, meat extract and casein hydrolysate.; **N.A.** = None added.; ****Electron acceptors: O2** = aerobic conditions; **NO_3^-/ NO_2^-** = anaerobic conditions with nitrate/nitrite added; **N.A.** = None added, anaerobic conditions. Where multiple electron acceptors are listed these were assessed with the electron donor separately.

Results and Discussion

MAR signals were not observed to be above background levels (anaerobic incubation with $^{14}CO_2$ alone) for the A21b and ABS-19 phylotypes, for any of the conditions tested (e.g. **Figure Ai. and ii.**). Although many MAR-positive non-target cells were observed for all conditions (see **Figure A**), without any positive signal for these target phylotypes it is difficult to conclude anything from these negative results; especially given signal for aerobic HetCO_2 assimilation for the *Sulfuritalea* spp. was assessed to be negative, despite observed positive aerobic uptake of amino acids for the genus. Increasing the emulsion exposure time from 10 to 15 days only served to increase the background.

Visual assessment of the *Sulfuritalea* spp. also indicated that most conditions gave negative $^{14}CO_2$ assimilation. Low activity was observed under thiosulphate oxidising conditions (**Figure Aiii.**), so semi-quantitative MAR (MARQuant) was applied to assess the significance. However, the results were inconsistent due to the low range of activity (data not shown).

The results of this work indicate that the sensitivity of $^{14}CO_2$-based MAR, as applied here, was not high enough for all species. The lower signal likely relates to the size and activity of the cells. The phylotypes analysed in this study were all relatively small single cells, where the technique has predominantly been applied to assess filamentous [1] and microcolony-producing organisms [4] where positive signal would be easier to visualize, acknowledging that positive single cells were detected in this study (see **Figure A**). Activity levels of the cells may be reduced by the preparation steps, such as lowering the pH to 5, or a failure to replicate the optimal

conditions experienced by the species in the full-scale sample. Therefore, the technique needs to be optimized to achieve the sensitivity required for these, and similar, phylotypes.

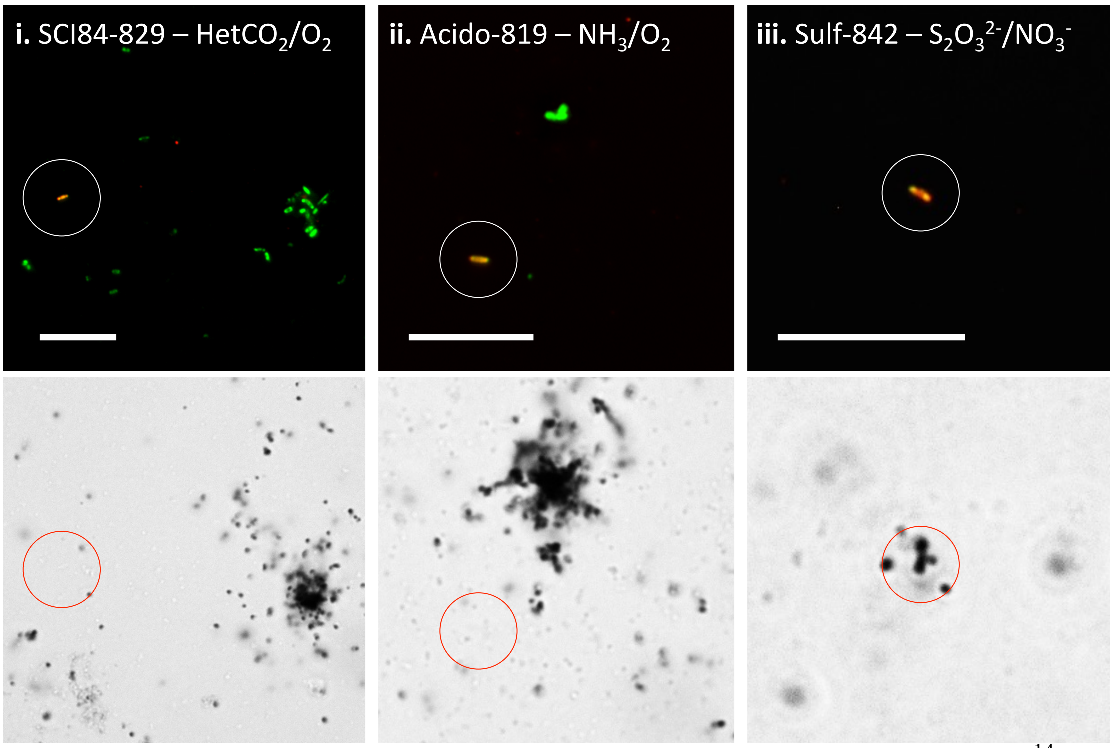

Figure A. FISH and corresponding bright-field MAR micrographs for $^{14}CO_2$ incubations. FISH probes and MAR electron acceptor/donor combinations are indicated for each image set. $HetCO_2$ = heterotrophic MAR were unlabeled complex carbon mix is included. Target cells in FISH micrograph overlays appear yellow [target probe (Cy3 = red) + EUBmix (FLUOS) = green] and non-target green (EUBmix only). Black silver granules indicate positive MAR signal. Circles indicate location of FISH-positive cells. Scale bars represent 10 µm.

References

1. Hesselsø M, Nielsen JL, Roslev P, Nielsen PH. Isotope labeling and microautoradiography of active heterotrophic bacteria on the basis of assimilation of $^{14}CO_2$. Appl Env Microbiol. 2005; 71: 646–655.

2. Morgan-Sagastume F, Nielsen JL, Nielsen PH. Substrate-dependent denitrification of abundant probe-defined denitrifying bacteria in activated sludge. FEMS Microbiol Ecol. 2008; 66: 447–461.

3. Nierychlo M, Nielsen JL, Nielsen PH. Studies of the ecophysiology of single cells in microbial communities by (quantitative) Microautoradiography and Fluorescence In Situ Hybridization (MAR-FISH). In: McGenity TJ, Timmis KN, Nogales Fernández B, editors. Hydrocarbon and Lipid Microbiology Protocols, Springer Protocols Handbooks. 1st ed. Berlin-Heidelberg: Springer-Verlag; 2015. doi:10.1007/8623_2015_66

4. Daims H, Nielsen JL, Nielsen PH, Schleifer KH, Wagner M. *In situ* characterization of Nitrospira-like nitrite-oxidizing bacteria active in wastewater treatment plants. Appl Env Microbiol. 2001; 67: 5273–5284.
